# Understanding Residents’ Physical Activity Intention and Behavior Amid COVID-19 Pandemic

**DOI:** 10.3389/fpsyg.2022.760702

**Published:** 2022-02-25

**Authors:** Xiaoyu Cheng, Wei Chen

**Affiliations:** College of Physical Education, Wuhan Sports University, Wuhan, China

**Keywords:** theory of planned behavior (TPB), physical activity, intention, behavior, global public health event, COVID-19

## Abstract

Proper and regular physical activity (PA) plays an important role in improving people’s health. With the outbreak of the COVID-19 pandemic, which has posed a serious threat to individual health, residents’ PA has aroused deep concern. Based on the theory of planned behavior, this study examines the impact of residents’ PA intention and behavior in response to the COVID-19 pandemic. Data were collected from a sample of 961 residents in Wuhan in China using a questionnaire survey. The results show that residents’ PA intention and behavior have been affected significantly by residents’ PA attitude, subjective norms, and perceived behavioral control. The COVID-19 pandemic has a significant impact on both residents’ PA intention and behavior. In addition, there were gender and region differences in this impact. The findings are of great significance in promoting residents’ PA intention and PA behavior, which are of positive significance to the development of residents’ physical and mental health in the period of global serious health crisis.

## Introduction

Previous studies have shown that proper and regular physical activity (PA) could not only maintain physical health but also help people mediate anxiety and depression, release pressure, and improve the immune system to prevent the serious complications of pandemic (Mahalakshmi et al., 2020; Marchant et al., 2021). PA is defined as any bodily movement produced by skeletal muscles that require energy expenditure, and the popular forms include cycling, sports, and play (World Health Organization [WHO], 2021a).

So far, the COVID-19 pandemic has posed a serious threat to individual health, which has led to more than 200 million confirmed cases and 5 million deaths (World Health Organization [WHO], 2021b). It has dramatically threatened not only people’s health but also mental health and cognitive functioning of people, such as increasing the risks of Alzheimer’s disease, language fluency, and so on (Lardone et al., 2021). International organizations and countries have implemented measures and introduced guidelines to promote and foster the habit of people’s PAs, especially in the period of self-isolation at home. WHO has advocated that it is important for residents to be as physically active as possible and has instructed the children aged 5–17 to play indoor games (e.g., jumping rope, high jump, and lifting weights) for at least 60 min a day and 3 days a week, and adults could do moderate-intensity indoor PA by climbing stairs, doing housework, dancing, and so on (World Health Organization [WHO], 2020a). There is increasing global concern and advocacy for people to take regular and proper PAs (Brand et al., 2020). Whether people’s PA intention and behavior have been influenced by the COVID-19 pandemic needs to be explored.

Studies have found that PA behavior is significantly affected by the social environment, individual intention, and other personal characteristics (Van Luchene et al., 2021). In addition, the intention of PA plays an essential role in predicting PA behavior (Cheon et al., 2012). Previous studies have applied the theory of planned behavior (TPB) to study an individual’s intention and behavior (Sarker et al., 2021). Ajzen (1985) suggested TPB based on the theory of rational behavior (TRA), which believed that an individual’s behavioral intention (INT) and behavior would be influenced by behavioral attitude (ATT), subjective norms (SNs), and perceived behavioral control (PBC; Ajzen, 1985; Venkatesh et al., 2000). Ajzen (2015) further elaborated the utility of TPB, that is, TPB is not a theory of changing behavior but is aiming to help explain and predict people’s intentions and behaviors (Ajzen, 2015). Behavioral intention, which refers to the subjective degree of an individual’s willingness to complete the behavior, could be measured to predict the occurrence of an individual’s behavior (Fishbein and Ajzen, 2010). The stronger the intention of an individual, the more the actual behavior reflects on the cards (Davis, 2000). Attitude refers to the individual’s favorable or unfavorable evaluation of the behavior (La Barbera and Ajzen, 2020). SN refers to the social pressure that a person feels about whether to take a kind of behavior, which includes normative belief and compliance motivation, namely, the individual’s view and willingness to follow the opinions of important people (Ajzen, 1991). Perceived behavioral control is an individual’s evaluation of influencing factors (such as experience, opportunities, and resources) that may advance or impede the behavior (Yadav and Pathak, 2017). It has been found that, when individuals experience more opportunities, resources, and fewer obstacles than expected, the level of PBC will increase (Barki, 1994). The more positive the attitude of individual behavior and the stronger the SNs and PBC ability are, the stronger the intention to perform behavior will be (Cheon et al., 2012).

Based on TPB, this study focuses on residents’ PA intention and PA behavior and their influencing factors before and after the outbreak of the COVID-19 pandemic to understand the impact of the COVID-19 pandemic on residents’ PA intention and behavior. In this study, Wuhan in China, which is one of the earliest outbreak cities of the COVID-19 pandemic and the earliest city to implement “lockdown” with suspending all the traffics, shops, factories, and schools on January 23, 2020, has been chosen to be the research region. The city has been unsealed since April 8, 2020, with the COVID-19 pandemic under control. In addition, the residents in Wuhan, which refer to those who have resided in Wuhan for at least 1 year from 2019, had gone through the most impressive experience of influences of the COVID-19 pandemic, especially self-isolation at home. This study puts forward the research hypotheses H1–H5 as follows.

H1: Residents’ PA intention has a significantly positive impact on residents’ PA behavior.

H2: Residents’ PA attitude, SN, and PBC have significantly positive impacts on residents’ PA intention.

H3: Residents’ PA intention has been significantly influenced by the COVID-19 pandemic.

H4: Residents’ PA behavior has been significantly influenced by the COVID-19 pandemic.

H5: There are gender and urban—rural differences in residents’ PA intention and behavior under the influence of the COVID-19 pandemic.

## Materials and Methods

### Measurement

#### The Theory of Planned Behavior Scale

Residents’ PA intention, attitude, SN, and PBC are measured based on the TPB-related structure scale developed by Ajzen (1991). The intention of PA is measured using three items, including “*I plan/intend/hope to do physical activity for at least 20 min at least three times a week in the next 2 weeks*.” The attitude of PA is measured by five items, including “*It is satisfying/pleasant/enjoyable/useful/important to me at least 20 min of physical activity at least three times a week for the next 2 weeks*.” SNs are measured by three items, including “*Most people who are important to me will happy when I do physical activity in the next 2 weeks, for more than three times a week, each time for at least 20 min*,” “*Most people who are important to me approve of me doing the above physical activity*,,” and “*Most people who are important to me hope me to do physical activity*.” PBC of PA is measured by four items, namely, “*It is easy for me to do the above physical activity*,” “*I can accept myself to do the above physical activity*,” “*It is beyond my ability to do physical activity*”, and “*I can control myself to do physical activity*.”

The internal consistency of the original measurement was 0.85 (Ajzen, 1991). All items adopted Likert’s 5-point scale from “*strongly disagree*” 1 to “*strongly agree*” 5. The original questionnaire was translated into Chinese by 4 bilingual scholars with more than 5 years of experience in translation and cross-cultural identification to ensure consistency between the two translated versions. The validity was evaluated by 10 experts, and a preliminary study was conducted on 30 volunteer participants. Based on the preliminary test, the questionnaire was modified to clarify the ambiguity.

#### The Physical Activity Rating Scale (PARS-3)

The PARS-3 proposed by Deqing (1994) was used to assess the PA rating of residents, and its internal consistency was 0.82, which passed the reliability and validity test (Deqing, 1994). Residents’ PA level is measured from three dimensions, intensity, duration, and frequency. Intensity is including “*light physical activity intensity*”, “*light physical activity with not too intense intensity*”, “*moderate and mild physical activity with more intense and persistent intensity*”, “*heavy but not persistent physical activity with shortness of breath and sweating a lot*”, and “*heavy and persistent physical activity with shortness of breath and sweating a lot*”. Duration level is divided as ≤10, 11–20, 21–30, 31–59, and ≥60 min, respectively. Frequency includes “*once a day*”, “*3–5 times a week*”, “*1–2 times a week*”, *“2–3 times a month*”, and “*once a month or less*”. The three dimensions of PARS-3 just meet *The WHO Guidelines on physical activity and sedentary behavior*, which provide recommendations for people on the amount of PA (frequency, intensity, and duration) (World Health Organization [WHO], 2020b).

A Likert-type 5 point scoring method was adopted. The total score of residents’ PA level was calculated using the following formula: *PA level* = *Intensity* × *(Duration-1)* × *Frequency* (Deqing, 1994). The evaluation criteria for light, moderate, and vigorous levels of PA is the total score ≤19, 20–42, and ≥43 points, respectively.

### Data Collection and Procedures

To understand the changes in PA behavior of Wuhan residents, data were collected two times: the first time before the pandemic outbreak (June 1, 2019–June 30, 2019) and the second time after the outbreak and lockdown of the COVID-19 pandemic (June 1, 2020–June 30, 2020) in the same community. Our sample included only residents aged 5 or older who claimed to reside in Wuhan during the period from June 2019 to June 2020. A total of 1,053 responses were collected with an effective rate of 91.3%, in which 92 responses were invalid and 961 responses were valid. Questionnaires were issued anonymously, and the content of the questionnaires was strictly confidential and can be used only for academic research. In the survey, 53.3% of residents were men and 46.7% were women, with ages 5–17, 18–34, 35–59, and 60 and above as 11.3, 26.5, 39.4, and 22.7%, respectively. The proportion of urban and rural residents was 50.2 and 49.8%, respectively (refer to Table 1).

**TABLE 1:** Demographic characteristics.

Characteristics	Sub-character	N (961)	%(100)
Gender			
	Male	512	53.3
	Female	449	46.7
Age-group (years)			
	5–17	109	11.3
	18–34	255	26.5
	35–59	379	39.4
	60 +	218	22.7
Region			
	Urban	575	59.8
	Rural	386	40.2

Stata 16.0 was used to study the relationship and changes in the PA intention and behavior of residents in Wuhan before and after the COVID-19 pandemic. Statistical analysis was conducted, and when *p* ≤ 0.05, the result was considered statistically significant (Alsalhe et al., 2020).

## Results

### Assessment of the Measurement Model

Cronbach’s alpha (α) and composite reliability (CR) were adopted to examine reliability (Nunnally, 1967; Bagozzi, 1981), and the values α> 0.7 and CR > 0.7 represent a commonly accepted level. As shown in Table 2, α values for all the scales exceeded 0.7, and the results showed that all the scales had good internal consistency. The result of KMO = 0.95 > 0.7 and *p* = 0.000 indicated that the variable can conduct factor analysis. The CR values exceeding 0.7 indicated that all the scales had good composite reliability.

**TABLE 2:** Reliability, convergent, and discriminant validities.

		α	CR	AVE	INT	ATT	SN	PBC	PA	M ± SD
Before	INT	0.946	0.9511	0.8675	**0.9314**					4.143 ± 0.970
	ATT	0.960	0.9601	0.8279	0.831***	**0.9109**				4.165 ± 0.884
	SN	0.935	0.9356	0.8289	0.712***	0.854***	**0.9104**			4.239 ± 0.851
	PBC	0.770	0.8238	0.5846	0.693***	0.791***	0.746***	**0.7646**		3.870 ± 0.779
	PA	0.762	0.7614	0.5159	0.383***	0.364***	0.302***	0.359***	**0.7183**	32.787 ± 26.095
After	INT	0.952	0.9564	0.8805	**0.9383**					4.1079 ± 1.001
	ATT	0.956	0.9568	0.8159	0.841***	**0.9033**				4.1863 ± 0.885
	SN	0.948	0.9485	0.8600	0.773***	0.888***	**0.9274**			4.207 ± 0.900
	PBC	0.771	0.8234	0.5826	0.702***	0.790***	0.758***	**0.7633**		3.881 ± 0.790
	PA	0.741	0.7626	0.5172	0.419***	0.435***	0.413***	0.438***	**0.7192**	32.624 ± 25.354

***p < 0.001. The thickened part of the diagonal is the square root of AVE. The lower triangle is the Pearson correlation coefficient. INT, behavioral intention; ATT, attitude; SN, subjective norm; PBC, perceived behavioral control; PA, physical activity.

The test of validity included convergence validity and discriminant validity (Segars, 1997). Standardized path loading (>0.7) and average variance extracted (AVE) (>0.5) were used to test convergence validity. All the values pass the test. Discriminant validity was tested by examining the correlation between the square root of the AVE and the other factors. The square roots of AVEs of scales indicate a good discriminant validity (refer to Table 2).

### Independent Samples *t*-Test

In the *t*-test analysis, only gender and urban-rural demographic variables were significant. Therefore, this study compared the differences in PA in gender and urban-rural before and after the pandemic. Gender was divided into two groups, namely, male and female, to study the difference in PA. The homogeneity of variance test showed that data of the two groups before and after the lockdown had homogeneity of variance (*F*_*before*_ = 0.002, *p* = 0.964 > 0.05, *F*_*after*_ = 0.251, *p* = 0.616 > 0.05). The *t*-test results showed *t*(df)_*before*_ = 3.424(959), *p* = 0.001 < 0.05, *t*(df)_*after*_ = 2.473(959), *p* = 0.014 < 0.05. Both before and after the pandemic, the scores of male residents’ PA was higher than that of female residents. However, after the pandemic, the difference in the score between male and female residents was smaller. The scores of male residents’ PA declined, while the scores of female residents’ PA increased. The region was divided into two groups to study the difference in PA between urban and rural. The homogeneity of variance test showed that data of the two groups before and after the pandemic had homogeneity of variance (*F*_*before*_ = 0.478, *p* = 0.489 < 0.05, *F*_*after*_ = 0.047, *p* = 0.829 > 0.05). The *t*-test results showed *t*(df)_*before*_ = −3.317(826.473), *p* = 0.001 < 0.05, *t*(df)_*after*_ = −1.472(959), *p* = 0.141 > 0.05. Both before and after the pandemic, the scores of rural residents’ PA were higher than that of urban residents. However, after the lockdown, the difference between urban and rural scores was smaller. The scores of rural residents’ PA declined, while the scores of urban residents’ PA increased (refer to Table 3). H5 has been verified.

**TABLE 3:** Independent samples *t*-test.

	Gender	M ± SD	Homogeneity test of variance		Independent samples *t*-test	
			*F*	*p*	*t* (df)	*P*
Before	Male	35.471 ± 25.821	0.002	0.964	3.424 (959)	0.001
	Female	29.726 ± 26.097				
	Urban	30.511 ± 25.973	0.478	0.489	−3.317 (826.473)	0.001
	Rural	36.176 ± 25.941				
After	Male	34.514 ± 24.758	0.251	0.616	2.473 (959)	0.014
	Female	30.470 ± 25.876				
	Urban	31.638 ± 25.618	0.047	0.829	−1.472 (959)	0.141
	Rural	34.093 ± 24.916				

### Regression Analysis

The behavioral intention was considered as the dependent variable, and attitude, SN, and PBC were considered as independent variables of multiple regression analysis. The P-P diagram and scatter diagram showed that the residual was approximately normal distribution with no heteroscedasticity problem. Variance inflation factor (VIF) was used to determine the collinearity of independent variables. Myers (1990) believed that VIF > 10 indicated strong collinearity (Myers, 1990). The results showed that there was no serious collinearity among independent variables. The variance interpretation rates of behavior intention by independent variables before and after the pandemic were as high as 69.5% and 71.4%, respectively, and the regression model fitted well (*F*_*before*_ = 726.004, *p* < 0.001, *R*^2^ = 0.695, *F*_*after*_ = 794.623, *p* < 0.001, *R*^2^ = 0.714) (refer to Table 4).

**TABLE 4:** Multiple linear regression analysis.

		Unstandardized coefficients		Standardized coefficients	*t*	*p*	VIF
		*B*	SE				
Before		0.725	0.282		2.576***	0.010	
	ATT	0.506	0.025	0.768	20.062***	0.000	1.261
	SN	−0.018	0.040	−0.016	−0.447	0.655	1.038
	PBC	0.090	0.028	0.097	3.234**	0.001	1.325
	R^2^			0.695			
	adj R^2^			0.694			
	F			726.004***			
After		−0.073	0.272		-0.268	0.789	
	ATT	0.465	0.028	0.685	16.788***	0.000	5.557
	SN	0.115	0.043	0.103	2.685**	0.007	4.909
	PBC	0.078	0.027	0.083	2.862**	0.004	2.778
	R^2^			0.714			
	adj R^2^			0.713			
	F			794.623***			

Dependent variable: physical activity intention. Independent variable: physical activity attitude (ATT), physical activity subjective norm (SN), physical activity perceived behavioral control (PBC). ***p < 0.001, **p < 0.01, *p < 0.05.

Behavior as the dependent variable and intention as the independent variable were considered for the simple linear regression analysis. The P-P graph and scatter graph were used to test the normal distribution and heteroscedasticity. It was proved that the residual of the unary regression model was close to the normal distribution with no heteroscedasticity problem, and the VIF test (VIF = 1.000) was passed. The regression model was verified to have a good fit (*F*_*before*_ = 164.600, *p* < 0.001, *R*^2^ = 0.146, *F*_*after*_ = 203.806, *p* < 0.001, *R*^2^ = 0.175) (refer to Table 5).

**TABLE 5:** Simple linear regression analysis.

		Unstandardized coefficients		Standardized coefficients	*t*	*p*	VIF
		B	SE				
Before		−9.875	3.415		−2.892**	0.004	
	INT	3.432	0.267	0.383	12.83***	0.000	1.000
	R^2^			0.146			
	adj R^2^			0.146			
	F			164.600***			
After		−10.928	3.140		−3.48**	0.001	
	INT	3.534	0.248	0.419	14.276***	0.000	1.000
	R^2^			0.175			
	adj R^2^			0.174			
	F			203.806***			

Dependent variable: physical activity behavior. Independent variable: physical activity intention (INT). ***p < 0.001, **p < 0.01, *p < 0.05.

Residents’ behavior intention had a statistically significant impact on the behavior of PA before and after the pandemic (*B*_*before*_ = 3.432, *t*_*before*_ = 12.83, *p* < 0.001; *B*_*after*_ = 3.534, *t*_*after*_ = 14.276, *p* < 0.001). The following regression equation was used: PA_*before*_ = 3.432 * INT-9.875, PA_*after*_ = 3.534 * INT-10.928. In other words, when the intention increases by 1 unit, the behavior will increase by 3.432 and 3.534 units, respectively. When the intention increases to a certain extent (INT_*after*_ > INT_*before*_ > 10.324, PA_*after*_ > PA_*before*_), the behavior of PA after the pandemic increases significantly compared with that before the pandemic. In terms of the PA level, the moderate level of PA increased slightly from 32.5 to 32.8%, the low level of PA decreased slightly from 30.1 to 29.8%, and the vigorous level of PA did not change significantly (37.5%). In addition, the results showed that the amount of residents’ PA (32.62 ± 25.35) reduced before the pandemic (32.79 ± 26.10), and the average amount of residents’ PA after was 0.17, which was lower than that before the pandemic. Specifically, after the pandemic, residents’ PA intensity decreased (*M*_*before*_ = 3.01 > *M*_*after*_ = 2.99), while the frequency (*M*_*before*_ = 3.67 < *M*_*after*_ = 3.68) and duration (*M*_*before*_ = 3.46 > *M*_*after*_ = 3.50) increased slightly. Hypothesis 1 and 3 were verified.

Among the three included independent variables, before the pandemic, residents’ PA attitude (*B* = 0.506, *t* = 20.062, *p* < 0.001) and PBC (*B* = 0.090, *t* = 3.234, *p* < 0.01) had statistically significant effects on intention, and the regression equation was INT = 0.506 * ATT-0.018 * SN + 0.09 * PBC + 0.725. After adjusting the collinearity changes of attitude, PBC, for every unit increase of attitude and PBC, residents’ intention before and after the lockdown increased by 0.506 and 0.09 units, respectively. After the pandemic, attitude (*B* = 0.465, *t* = 16.788, *p* < 0.001), SN (*B* = 0.115, *t* = 2.685, *p* < 0.01), and PBC (*B* = 0.078, *t* = 2.862, *p* < 0.01) had statistical significance on intention. The regression equation was INT = 0.456 * ATT + 0.115 * SN + 0.078 * PBC-0.073. After adjusting the collinearity of attitude, SN, and PBC, residents’ intention before and after the pandemic intention increased by 0.456, 0.115, and 0.078 units, respectively. Hypothesis 2 and 4 were verified.

## Discussion

In summary, the main findings of this study were as follows: (i) whether before or after the pandemic, residents’ PA intention has a significantly positive impact on PA behavior; (ii) residents’ PA behavior has decreased after the outbreak of the COVID-19 pandemic and there are notable gender and region differences in the aspect of physical activity behavior of residents; and (iii) there are significant changes in residents’ PA intention due to the influence of the COVID-19 pandemic. Before the pandemic, residents’ PA attitude and PBC have a significantly positive impact on residents’ PA intention. However, after the pandemic, besides residents’ PA attitude and SNs, the PBC has a significantly positive impact on residents’ PA intention.

Whether before or after the pandemic, residents’ PA intention has a significant impact on PA behavior. Studies have confirmed that the outbreak of pandemics did reduce residents’ PA (Brand et al., 2020; Castaeda-Babarro et al., 2020). According to the results of the correlation of PA intention and behavior, it is possible to have more physical activities only if the intention is stronger. Male residents do more PA than female residents, which has been proved by previous research (DeWolfe et al., 2020). Men are more likely to participate in physical activities in venues, such as football, but due to the lockdown, various venues have been closed, which has greatly restricted the places where men used to do PAs (Christofaro et al., 2021). Unlike men, women do more work in domestic places, which may account for the increase in PA (Armstrong et al., 2006).

Residents’ PA intention is affected by attitude, PBC, and SNs. An interesting finding is that before the pandemic, residents’ PA, attitude, and PBC had a significant impact on residents’ PA intention, in which PA attitude has a greater impact on residents’ PA intention, while SNs have no significant impact. This is consistent with the existing studies that residents’ positive or negative evaluation of participating in PA, as well as their perception of their ability, experience, and opportunity to participate in PA, will affect their PA intention and behaviors (Marashi et al., 2019; Alipour-anbarani et al., 2021). After the outbreak of the pandemic, the influence of residents’ PA attitude and PBC on residents’ PA intention are slightly weakened and residents’ PA attitude is still the most significant factor influencing residents’ PA intention. However, it is worth noting that SNs have a significant impact on residents’ PA intention, and the impact is greater than the PBC. The weakened predictive effect of residents’ PBC on PA intention and behavior may be because residents experience more obstacles when attempting PA (such as reduced opportunities, scarce knowledge, and experience in PA, or beyond their own capabilities) (Ajzen, 1991; Yadav and Pathak, 2017; Wallace, 2020). However, the support and affirmation of important family members and friends, the promotion of policies, and the vigorous publicity of the government make residents feel unprecedented social pressure, thus affecting residents’ willingness to participate in PAs and increasing residents’ PAs. During the lockdown, the government in Wuhan and China have issued accurate guidelines on conducting PAs for residents, updating not only the new standards for residents’ participation in PA but also the types of PA (such as yoga and aerobics). These led residents’ SNs to constantly strengthen despite the weakening of attitude and perceived behavior control. *The outline of the “Healthy China 2030” plan* published by the government of China calls for a wide range of public participation in PA with specific requirements such as ensuring that students should participate in PA for more than 1 h at school and participate in PA at moderate intensity at least three times a week (Central People’s Government of the People’s Republic of China, 2016). The government of Wuhan has launched a *home scientific fitness guide*, which suggests children practice physical activities indoors such as standing on one foot or crawling movement; teenagers do 1 h indoor PA every day, such as fitness and aerobics; healthy adults do 150 min of indoor PAs at least 3 days a week, such as walking, jogging, or Qigong; and the elderly do 150 min of indoor PAs, such as tai chi or softball, at least 3 days a week (Wuhan Sport Bureau, 2020).

## Conclusion and Recommendations

From what has been discussed above, residents’ PA intention and behavior have been significantly influenced by the COVID-19 pandemic, and residents’ PA intention has been directly influenced by residents’ PA attitude, PBC, and SNs. Although the residents’ PBC has decreased, the function of SNs comes into play.

According to the study results, multiple and effective measures need to be put forward. First, guiding residents to form more positive attitudes, SNs, and stronger PBC. Second, promoting health education and publicity of the importance and usefulness of PA to guide residents to form correct cognition and positive attitude by international organizations and governments. Third, strengthening the role of the Internet through popularizing home PA guidelines, prompting online courses according to the needs of different groups, cultivating residents’ interest and confidence, and eventually guiding residents to form a PA habit. Last but not least, giving full play to the role of family through creating a good family atmosphere to guide family members to participate in PAs and enhancing family immunity.

The generalization of these results, which is subject to certain limitations, provides avenues for future research. One of the limitations in this study, which could have affected the measurements, was the tested sample. This study chose 961 residents in Wuhan as the research object group. Although the internal consistency fell above an acceptable level, the relatively small sample size used in the study cannot be a general adaption to the whole situation. Given this, studies could be carried out in a broader population. Moreover, more accurate PA measurement tools should be considered based on more critical appraisal and consideration within the limitations to increase the validity and reliability of studies. Third, more changing factors such as the types of PA practiced by residents could be considered to conduct further research.

Notwithstanding these limitations, the study has offered some insight into promoting residents’ PA intention and behavior, which are of positive significance to the development of residents’ physical and mental health in the period of global serious health crisis. This would be a fruitful area for further work.

## Data Availability Statement

The raw data supporting the conclusions of this article will be made available by the authors, without undue reservation.

## Ethics Statement

Ethical review and approval was not required for the study on human participants in accordance with the local legislation and institutional requirements. The questionnaire data were collected anonymously in this study, and the questionnaire filling was voluntary. The subjects were promised that the questionnaire data would only be used for academic research and kept strictly confidential. The participants provided their written informed consent to participate in this study.

## Author Contributions

XC: mainly responsible for data collection, analysis, and manuscript writing. WC: responsible for the revision, proofreading, and supervision of the study. Both authors contributed to the article and approved the submitted version.

## Conflict of Interest

The authors declare that the research was conducted in the absence of any commercial or financial relationships that could be construed as a potential conflict of interest.

## Publisher’s Note

All claims expressed in this article are solely those of the authors and do not necessarily represent those of their affiliated organizations, or those of the publisher, the editors and the reviewers. Any product that may be evaluated in this article, or claim that may be made by its manufacturer, is not guaranteed or endorsed by the publisher.
